# Recent advances in the application of gasotransmitters in spinal cord injury

**DOI:** 10.1186/s12951-024-02523-3

**Published:** 2024-05-23

**Authors:** Xiang Gao, Bingrong Jin, Xiaozhong Zhou, Jinyu Bai, Hao Zhong, Kai Zhao, Zongrui Huang, Chao Wang, Jiang Zhu, Qin Qin

**Affiliations:** 1 Department of Orthopedics, The Second Affiliated Hospital of Soochow University, Suzhou, 215000 Jiangsu China; 2 Department of Anesthesiology, The Second Affiliated Hospital of Soochow University, Suzhou, 215000 Jiangsu China; 3 Institute of Functional Nano & Soft Materials (FUNSOM), Jiangsu Key Laboratory for Carbon-Based Functional Materials and Devices, Soochow University, Suzhou, 215123 Jiangsu China

**Keywords:** Gasotransmitters, Oxidative stress, Spinal cord injury, Nerve regeneration, Nanocarriers

## Abstract

Spinal Cord Injury (SCI) is a condition characterized by complete or incomplete motor and sensory impairment, as well as dysfunction of the autonomic nervous system, caused by factors such as trauma, tumors, or inflammation. Current treatment methods primarily include traditional approaches like spinal canal decompression and internal fixation surgery, steroid pulse therapy, as well as newer techniques such as stem cell transplantation and brain-spinal cord interfaces. However, the above methods have limited efficacy in promoting axonal and neuronal regeneration. The challenge in medical research today lies in promoting spinal cord neuron regeneration and regulating the disrupted microenvironment of the spinal cord. Studies have shown that gas molecular therapy is increasingly used in medical research, with gasotransmitters such as hydrogen sulfide, nitric oxide, carbon monoxide, oxygen, and hydrogen exhibiting neuroprotective effects in central nervous system diseases. The gas molecular protect against neuronal death and reshape the microenvironment of spinal cord injuries by regulating oxidative, inflammatory and apoptotic processes. At present, gas therapy mainly relies on inhalation for systemic administration, which cannot effectively enrich and release gas in the spinal cord injury area, making it difficult to achieve the expected effects. With the rapid development of nanotechnology, the use of nanocarriers to achieve targeted enrichment and precise control release of gas at Sites of injury has become one of the emerging research directions in SCI. It has shown promising therapeutic effects in preclinical studies and is expected to bring new hope and opportunities for the treatment of SCI. In this review, we will briefly outline the therapeutic effects and research progress of gasotransmitters and nanogas in the treatment of SCI.

**Graphical Abstract:**

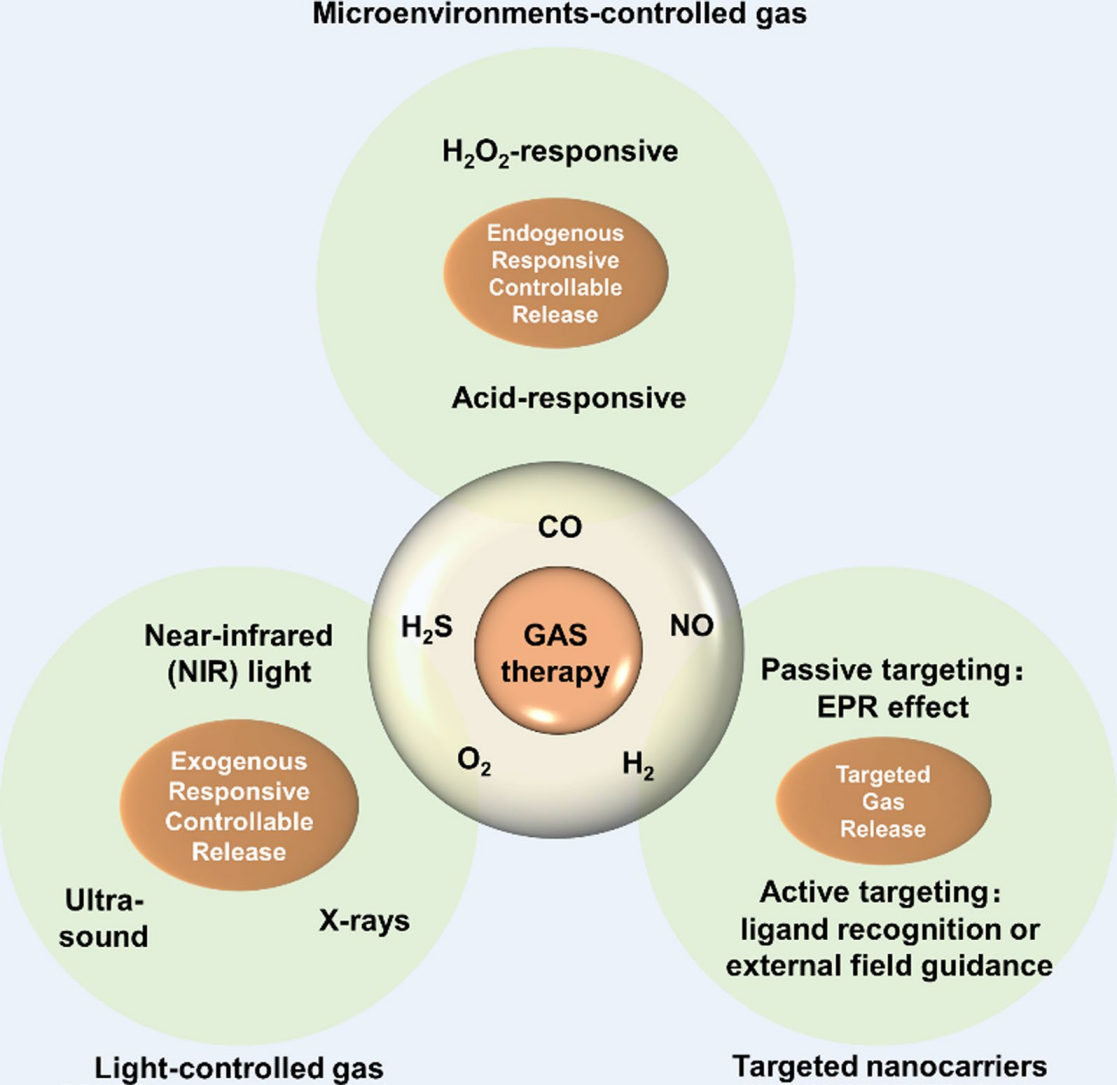

## Introduction

With the ongoing growth of the construction, transportation, and sports industries, the incidence of SCI has been steadily increasing, particularly among young men. Approximately 30 million individuals worldwide are currently living with SCI, and an additional 250,000–500,000 new cases emerge each year, imposing a significant economic burden on society [1]. SCI is categorized as primary or secondary based on the course of the disease. Primary SCI is the result of direct action by external forces, such as compression, tearing, acute stretching and distraction, which are neither unpredictable nor prevented Following the primary injury, a cascade of detrimental secondary pathological processes is triggered, including ischemia, apoptosis, necrosis, inflammatory response, edema, free radical damage, mitochondrial dysfunction, oxidative stress, and glial scar formation [2]. Although current dominant therapies for SCI include surgical decompression, hormonal shock therapy and stem cell transplantation, they are limited by efficacy and often associated with various complications, restricting the regression and prognosis of patients with SCI [3].

Gasotransmitters, comprising a group of small gaseous molecules that can freely pass through biological membranes, are essential compounds within the body.They are generated in vivo from specific substrates by rate-limiting synthetic enzymes and carry out physiological functions within a specific concentration range, which can be replicated by external donors [4]. Currently, hydrogen sulfide (H_2_S), nitric oxide (NO), and carbon monoxide (CO) are the recognized gasotransmitters that exert diverse biological functions through specific cellular and molecular targets. Moreover, recent researches have indicated that hydrogen (H_2_) and hyperbaric oxygen (HBO) also play crucial roles in the nervous system and are considered novel gasotransmitters [5]. Abnormal gas signaling pathways are indicative of the initiation and progression of inflammation and disease [6, 7]. Previous studies have demonstrated that gases such as H_2_S, NO, CO and H_2_ primarily possess anti-inflammatory and antioxidant properties and their beneficial biological effects can be harnessed to aid in the treatment of central nervous system-related conditions [8]. This review will introduce the application of different Gasotransmitters and nanogases in SCI diseases.

## Intervention and regulatory effects of hydrogen sulfide on SCI

H_2_S is a colorless gas with a characteristic rotten egg odor and has long been recognized as a toxic gas and environmental pollutant. Research indicates that H_2_S has participated in various physiological and pathological processes [9].H_2_S play an important role in the nervous system, cardiovascular system and immune system, and participates in a variety of cellular metabolic functions, including the regulation of mitochondrial function, glucose and lipid metabolism, oxidative stress response [9].

### The biosynthesis and functional roles of H_2_S

In the presence of cysteine reductants, endogenous H_2_S is generated through a direct enzymatic dehydration reaction catalyzed by cystathionine beta-synthase (CBS) and cystathionine gamma-lyase (CSE), as well as an indirect desulfuration reaction catalyzed by 3-mercaptopyruvate sulfurtransferase (3-MST). CBS is predominantly present in the central nervous system and liver, while CSE is mainly found in the cardiovascular system, and 3-MST is located in the mitochondria, working in coordination with cysteine aminotransferase (CAT) to produce H_2_S [10].

Research has found that H_2_S, similar to CO and NO, plays an important role in anti-inflammatory and anti-apoptotic effects in oxidative stress reactions [11, 12]. H_2_S and its donor, sodium hydrosulfide (NaHS), have protective effects on the spinal cord and related diseases [13, 14]. H_2_S is also effective in the treatment of SCI-induced complications, such as osteoporosis [15]. The lack of H_2_S can lead to defects in the osteogenic differentiation of bone marrow mesenchymal stem cells, while exogenous H_2_S inhibits the activity of osteoclasts, alleviating osteoporosis. Exogenous H_2_S increases the number of osteoblasts in the tibia, as well as the levels of osteocalcin in the serum and femur, promoting the recovery of osteoblast activity in SCI rats [16]. As an H_2_S donor, NaHS effectively reduces the degradation of Tight Junction(TJ) and Adherens Junction(AJ) proteins, inhibits endoplasmic reticulum stress and related autophagy, prevents an increase in Blood-Spinal Cord Barrier(BSCB) permeability, protects the spinal cord against secondary injury, and promotes the recovery of spinal cord function [17]. NaHS achieved its protective effect on SCI rats 1 h after clamping by reducing various oxidative indicators and increasing antioxidants and their regulators [18]. Additionally, H_2_S exerts anti-inflammatory, antioxidant and neuroprotective effects by activating the nuclear factor erythroid 2-related factor 2 (Nrf2) signaling pathway, increasing Nrf2 protein levels [19].

### H_2_S inhibits the immune-inflammatory response and promotes axon growth of neuronal cells

H_2_S has a protective effect on the nervous system, which can improve the symptoms of neuritis, reduce the secretion of inflammatory factors, nerve cell apoptosis and oxidative stress response, and protect neurons from secondary neuronal damage [20]. In the early stages, neutrophils (peaking at day 1 post-injury) and macrophage/microglia (peaking at day 7 post-injury) are the main components of the inflammatory response in SCI [21]. H_2_S as immune modulator promotes the expression of the anti-apoptotic B cell lymphoma-2 (Bcl-2) [22], inhibits the expression of the pro-apoptotic Bax protein (Bcl-2 Associated X), and reduces the release of interleukin-1 (IL-1) and tumor necrosis factor-α (TNF-α). At the same time, the release of IL-1 and TNF-α is reduced, ultimately leading to the weakening of the Nuclear Factor Kappa-B (NF-κB) pathway p65 and enhancing protein kinase B phosphorylation [23], thereby exerting anti-inflammatory effects. In addition, H_2_S promotes axon growth of neuron [24] and acts as neuroprotectant to treat spinal cord ischemia–reperfusion injury by inhibiting the miR-30c expression and activating autophagy proteins (Beclin-1 and LC3II) [25].

### H_2_S and donors exert protective effect in models of neurological disorders

Studies have shown that inhalation of H_2_S effectively prevents the degeneration of spinal motor neurons in the ventral horn of the spinal cord and delayed-onset paralysis in mice after transient spinal cord ischemia [26]. However, H_2_S gas is toxic, flammable, and explosive, and inhalation of H_2_S has drawbacks, mainly in terms of gas storage, safe drug delivery, and targeting. Compared with traditional anti-inflammatory drugs, H_2_S-releasing nonsteroidal anti-inflammatory drugs (ATB-346) produced a more significant therapeutic effect in restoring motor function and reducing inflammation and apoptosis after SCI [27]. Fe_3_S_4_ hydrogel releases H_2_S slowly at a concentration of 10 μM, exhibiting excellent anti-inflammatory and neurotrophic effects. The dual effects of immunomodulation and anisotropy of Fe_3_S_4_ FFH make it a promising drug candidate for the treatment of SCI [28]. The H_2_S sustained-release donor (ADT-OH) can promote self-renewal and anti-apoptotic capabilities of neural progenitor cells (NPCs), maintaining normal brain function. It is a promising pharmacological agent for regulating neurogenesis in NPCs, with potential for clinical research and application [29].

## Research Progress on the Protective Effect of Nitric Oxide on SCI

### Biosynthesis and Functional Roles of Nitric Oxide

NO as endogenous bioregulators, has been demonstrated to participate in physiological and pathological events in the central nervous system and other types of tissues. The synthesis of endogenous nitric oxide is regulated by nitric oxide synthase (NOS), which has three isoforms: Induction type (iNOS/NOS2), nervous system type (nNOS/NOS1), and endothelial type (eNOS/NOS3). NO reacts with oxygen as a radical to form peroxynitrite and other reactive nitrogen, which are involved in lipid peroxidation, demyelination, neuronal apoptosis, and oligodendrocyte loss, inducing oxidative damage and leading to neuronal loss [30]. Hamada et al. reported that NO produced by inducible nitric oxide synthase (iNOS) has neurotoxic effects [31], while NO produced by constitutive nitric oxide synthase (cNOS) has neuroprotective effects.

## Double-Edged Role of Different Typed Nitric Oxide in Neurological Diseases

iNOS is involved in delayed neuronal and glial cell death, and the inhibition of iNOS activity after SCI can alleviate secondary SCI neuronal apoptosis [32]. At 6 weeks post-SCI, the iNOS-CKO group shows less white and gray matter compared to the control group, with fewer axons and peri-lesional blood vessels in the injury area. Research has reported that photobiological regulation reduces the expression of iNOS and STAT3, promoting motor function recovery in mice with SCI [33]. Nanoparticles loaded with iNOS inhibitors effectively reduce spinal cord inflammation and oxidative damage, significantly restoring motor function in SCI rats [34].

cNOS is divided into neuronal nitric oxide synthase (nNOS) and endothelial nitric oxide synthase (eNOS). The level of NO produced by nNOS reaches its maximum immediately after SCI, about 5 times that of the uninjured spinal cord, and starts to decline after 12 h post-injury. From 24 h to 3 days post-injury, NO levels increase for the second time, approximately twice that of the control group. The NO content gradually decreases until 14 days post-injury [35]. nNOS causes neurotoxicity after cerebral ischemia through the strong oxidant peroxynitrite, inhibiting the improvement of neurological symptoms after SCI. During the subacute phase of mild SCI, eNOS is activated, leading to a significant increase in spinal cord blood flow at the site of injury, and participating in protective and repair responses [36]. Metformin promotes vascular regeneration in the injured spinal cord and improves neurological function in SCI mice by activating the AMP-activated protein kinase/eNOS pathway [37]. Studies in the SCI mice indicate that nNOS-CKO or iNOS-CKO improved motor recovery [38, 39].

A gene silencing strategy based on small interfering RNA (siRNA), siRNA-chitosan NPs, reduces the expression of iNOS in M1 macrophages after SCI and decreases NO production through high transfection efficiency [40]. This induces systemic depletion of L-arginine, resulting in a local decrease in arginine levels and a reduction in NO concentration, thereby reducing NO-mediated cytotoxicity and neuronal apoptosis [41]. Although there are substantial research on inhibition methods targeting iNOS induced NO production, NO also plays crucial physiological roles. Apart from its prominent role in regulating cerebral blood flow and intercellular communication in the brain, NO has been found to be an effective antioxidant. At present, it is believed that the neurotoxicity of SCI is associated with the accumulation of low concentrations of NO derivatives such as nitrate and nitrite, rather than NO [42].

## Research progress on the protective effects of carbon monoxide on SCI

### Biological synthesis and functional roles of carbon monoxide

CO as bio-signaling molecule that is produced in living organisms during the degradation of hemoglobin by heme oxygenase (HO). The average CO generation rate in the human body is approximately 20 μmol/h. HO is an inducible enzyme for endogenous CO, and its activity accounts for 80–86% of endogenous CO production [43]. Biochemically, HO belongs to the heat shock protein (HSP) family and has three isoforms: HO-1, HO-2, and HO-3 [44]. HO-1 and CO are important for the maintenance of endogenous homeostasis, messenger transduction, and cytoprotection, have desirable therapeutic value [45]. Once the organism is challenged by stress, such as inflammation, cells increase the production of HO and CO to restore homeostasis and protect tissues [46, 47]. HO-1 is a widely expressed inducible enzyme that degrades hemoglobin into CO, ferrous ions (Fe^2+^), and biliverdin [48].

Exogenous CO is typically considered a toxic gas with a high affinity for hemoglobin (Hb). It forms carboxyhemoglobin (COHb) by binding with Hb, which in turn impairs oxygen delivery to tissues. The action of CO is similar to NO, activating soluble guanylate cyclase and increasing the production of cyclic guanosine monophosphate (cGMP). It inhibits platelet aggregation, reduces leukocyte adhesion and cell apoptosis, and lowers the production of pro-inflammatory cytokines [48]. CO, as a product of heme degradation, has been shown to have various biological functions such as anti-inflammatory, anti-apoptotic and antioxidant at low concentrations [48, 49]. The safe dose of CO promotes neuro-regeneration by triggering the sGC/cGMP/MAPK signaling pathway and a cascade between the HO-CO, HIF-1α/VEGF, and NOS pathways [50, 51]. The safe dose of CO inhalation therapy has been shown to reduce cell death in various organs, including the brain, spinal cord, heart, retina, kidneys, and lungs [51–54]. It promotes functional recovery in models of trauma, cerebral ischemia, myocardial infarction, and ischemia–reperfusion injury [51–54]. Inhalation of CO successfully promoted the recovery of locomotor function in SCI rat [55]. Due to the non-tissue-specific nature of inhaling CO, a portion of CO is delivered in vivo through plasma and carboxyhemoglobin (COHb), leading to hypoxia and toxic reactions in some tissues. Therefore, novel systems capable of controlled delivery and release of CO are referred to as carbon monoxide-releasing molecules (CORMs) [56]. In addition to inhaled CO, CO can also be delivered in the form of exogenous CO donors such as CORM1 or CORM3 [57].

### Functional roles of carbon monoxide and carbon monoxide-releasing molecules in SCI

Mechanical shock to the spinal cord during SCI leads to vascular rupture and tissue destruction, which may subsequently increase hemoglobin production (from dead cells or Hb). Meanwhile, the expression and activity of HO-1 are upregulated compared to the uninjured spinal cord [58]. Studies indicate that SCI increases the expression of HO-2, inducing an increase in CO production, leading to cell damage [59]. Brain-derived neurotrophic factor (BDNF) has neuroprotective effects in SCI, and its mechanism involves attenuating CO production by downregulating the expression of HO-2 [60].

COHb was maintained at a safe concentration of 6% throughout the 24-day dosing period and remained stable via intraperitoneal injection of CORM-3 (40 mg/kg/day) [61].

Exogenous administration of CORM-3 increases the concentration of CO in spinal cord tissue and alleviates neuronal necrosis after SCI. The mechanism may be related to the regulation of the inflammasome signaling pathway mediated by inositol-requiring enzyme 1 (IRE1) [62]. Exogenous CO prevents the denaturation of tight junction proteins and infiltration of neutrophils through increased CORM-3, thereby inhibiting BSCB injury and promoting motor recovery after SCI [63]. CORM-3 is a potential therapeutic approach for the treatment of SCI by inhibiting inflammatory vesicle activation and pyroptosis in neurons, improving histopathological and functional outcomes, and attenuating neuronal death after SCI [57, 64]. The release half-life of CO is very short (about 1 min), and a solid lipid nanoparticle (CORM-2-SLN) containing CORM-2 has been developed to achieve slow release of CO, improve its solubility, and achieve good therapeutic results in BSCB disruption and endothelial cell death after SCI [65].

## Research progress on the protective effects of hyperbaric oxygen on spinal cord injury

### Biofunctional effects of hyperbaric oxygen

Hyperbaric Oxygen Therapy (HBOT) refers to the administration of pure oxygen at a pressure higher than one atmosphere for the treatment of various diseases such as SCI, Alzheimer's disease, cognitive improvement, diabetes, and hard-to-heal wounds [66–70]. Low-pressure, low-oxygen environments can have an impact on human systems such as the respiratory, circulatory and digestive systems and high concentrations of oxygen in the blood may decrease brain tissue hypoxia, thus preventing neuronal cell death [71]. Hypoxia leads to increased oxidative stress, resulting in the production of oxygen and nitrogen reactive free radicals, which are extremely toxic and lead to cellular damage, death, and apoptosis.

### Research on the application of hyperbaric oxygen in SCI

HBOT helps to correct the hypoxic environment by increasing oxygen delivery to improve antimicrobial activity and attenuating hypoxia-induced factors [71]. HBOT is commonly used in the early stages of trauma and has achieved effective therapeutic effects [69]. Administering HBOT immediately after SCI is effective and has antioxidative, anti-apoptotic, and anti-inflammatory effects [72, 73]. Meanwhile, HBOT can enhance neurological function recovery and early rehabilitation exercise after SCI. Its mechanism may involve regulating macrophage polarization, suppressing inflammation related to SCI rat, protecting neural function, and promoting muscle movement recovery [74]. HBOT increases the oxygen content in the blood on one hand and enhances the diffusion distance of oxygen on the other hand. Simultaneously, it dilates small arteries, and improves local microcirculation, thereby reducing the degree of ischemic-hypoxic injury and edema around the spinal cord [75]. HBOT increases the oxygen levels of tissues to promote capillary angiogenesis, reduce inflammatory responses in damaged tissues, and accelerate tissue healing [74].

HBOT reduces SCI-induced spinal cord edema, stabilizes BSCB, and promotes neurological recovery by up-regulating vascular endothelial growth factor (VEGF) and down-regulating IL-6, matrix metalloproteinase-2 (MMP-2), and MMP-9 [76]. HBOT reduces levels of spinal cord superoxide dismutase (SOD), glutathione peroxidase (GPX), NOS, and NO [77], thereby decreasing secondary damage caused by inflammatory responses and promoting neurological function repair [76]. Early administration of HBOT in SCI reduces the synthesis of IL-1β and TNF-α cytokines, decreases neuronal apoptosis and glial cell density in injured rats, and promotes functional recovery [78]. HBOT in combination with methylprednisolone, human placental mesenchymal stem cell-derived exosomes, or quercetin produces synergistic neuroprotective effects, reduces inflammation at the site of spinal cord trauma, improves motor function, and accelerates SCI healing [73, 79, 80]. HBOT promotes the recovery of sensory and motor functions in SCI [69, 81, 82], effectively inhibits the expression of monocyte chemotactic protein-1 (MCP-1) in the damaged spinal cord, reduces neutrophil infiltration and secondary inflammatory responses, and promotes neurological function recovery [83, 84].In the spinal cord tissue of the HBOT group in the SCI model, there is an increase in the expression of Bcl-2, accompanied by decrease in Bax levels and reduction in the number of apoptotic cells, contributing to the improvement of motor function in the SCI model [85].

### Functional role of hyperbaric oxygen in SCI complications

HBOT improves bone turnover index and promotes bone formation in SCI rats, and its mechanism is associated with improved morphology and biomechanical properties of bone trabeculae and collagen [86]. HBOT enhances oxidative capacity, reduces the accumulation of reactive oxygen species (ROS), maintains diaphragm muscle fiber size and contractility, and enhances respiratory function recovery after SCI [87]. HBOT is used for clinical degenerative disc disease, with anti-inflammatory and pain relief effects [88]. The latest clinical studies have shown that the neurocervical spine scale (NCSS) has improved significantly in the HBOT group in patients with cervical hyperextension SCI without fractures. HBOT also improved ASIA and Frankel grading, motor function, as well as psychological status among SCI patients [69, 89, 90].

## Research progress on the protective effect of hydrogen on SCI

### Functional roles of hydrogen gas

Hydrogen (H_2_) is a non-toxic, colorless, odorless, diatomic gas with minimum density and not easily soluble in water, as a newer therapeutic antioxidant with reducing properties. H_2_ is inexpensive, easy to prepare, safe to use, and has a wide range of applications in clinical therapy.

In recent years, H_2_ has been found to be a medical gas molecule with anti-inflammatory, antioxidant, and anti-apoptotic effects, which can easily pass through the blood–brain barrier and cells while scavenging free radicals in the body, and has shown good therapeutic effects in a variety of disease models and clinical trials [91–95]. H_2_ does not easily undergo chemical reactions with other substances, and its properties are stable at room temperature. However, when conditions are altered, such as using a catalyst or heating, it can undergo corresponding chemical reactions. Extensive research and experiments have not found hydrogen to be toxic to the human body. Currently, the methods of using H_2_ can be broadly categorized as follows: inhalation of H_2_, drinking of H_2_-dissolved water, intraperitoneal or intravenous injection of hydrogen-rich physiological saline. It can also produce H_2_ by inducing large intestinal bacteria in vivo and play a corresponding therapeutic effect H_2_ is currently showing favorable results in the treatment of many diseases such as diabetes, sepsis, atherosclerosis, hypertension, cancer and other diseases [96–98].

### Study on the function of hydrogen in SCI

In the rat cerebral ischemia–reperfusion model, H_2_ selectively eliminates ROS and exhibits a protective effect [99]. In the rabbit spinal cord ischemia–reperfusion model, H_2_ increases the activities of catalase and superoxide dismutase, reduces cell apoptosis, improves the pathological features of spinal cord tissue, and increases the number of motor neurons [100]. High concentrations of H_2_ produce a neuroprotective effect by reducing ROS production, mitigating mitochondrial damage, and inhibiting cell apoptosis [101]. Both inhalation of H_2_ and intraperitoneal injection of saturated hydrogen solution have a protective effect on spinal cord ischemia–reperfusion injury [100, 102]. This effect is associated with a significant increase in BDNF levels and a decrease in oxidative stress levels [103]. Inhalation of H_2_ has a concentration-dependent protective effect on ischemia–reperfusion injury in the spinal cord. This may be achieved by increasing the expression of glutamate transporter-1 and inhibiting extracellular glutamate to reduce neuronal damage [104]. The latest study found that H_2_ significantly enhanced the therapeutic effect of bone marrow mesenchymal stem cells (BMSC) on SCI, promoted the migration and proliferation of BMSC, and repaired SCI [105].

Patients with SCI often experience residual sensory and motor function impairments. Activation of astrocytes post-SCI impedes axonal regeneration, while scar formation from proliferating and activated astrocytes inhibits their repair function to some extent. In the SCI rat, intraperitoneal injection of hydrogen-rich saline is found to reduce the production of ROS and improve locomotor scores [103]. Additionally, the levels of IL-6, TNF-α, and the quantity of apoptotic cells in the hydrogen-rich saline treatment group are significantly lower than those in the SCI group [106]. Moreover, after the injection of hydrogen-rich saline into the subarachnoid space, there is a significant decrease in inflammatory cell infiltration, serum malondialdehyde levels, serum SOD activity, and immunoreactivity of calcitonin gene-related peptides. This leads to an improvement in motor function [107]. Hydrogen-rich saline alleviates spinal cord ischemia–reperfusion injury by activating the mitochondrial ATP-sensitive potassium channels(mitoK_ATP_), reducing oxidative stress, inflammatory cytokines, and cell apoptosis. It promotes neuronal survival post-SCI and enhances the regeneration of injured axonal myelin sheaths by increasing BDNF [102, 103]. Reducing the release of reactive oxygen species(ROS) and related pro-inflammatory factors, inhibiting excessive proliferation of astrocytes post-SCI, and protecting neuronal growth by suppressing the formation of glial scars [108].

## The application of nanotechnology therapy in SCI

At the present stage, the clinical gas therapy is mainly carried out by inhalation, which is easy to induce body poisoning and difficult to realize the on-demand gas release in the lesion area, seriously restricting the applied range [109]. In recent years, the rapid development of nanotechnology has laid the foundation for precise delivery and controlled release of gases in vivo, providing a new perspective for precise gas therapy [110, 111]. The application of nanocarrier in nerve-related diseases is now reviewed to provide ideas for the clinical translational application of gas therapy.

### Exogenous responsive controllable release of gases

Among exogenous sources of stimulation, light-controlled gas release is a common route of controlled drug delivery. Most photoresponsive Gase Releasing Molecule (GRMs) prodrugs are sensitive only to UV or visible light [112, 113].The limited tissue penetration depth of UV and visible light and its tendency to cause phototoxicity severely limit the use of photoresponsive gas release in vivo. Building upon this, near-infrared (NIR) light has greater tissue penetration depth and lower phototoxicity. Even NIR light itself has a promoting effect on the recovery of motor function and neural regeneration after spinal cord injury [114]. NIR light-responsive gas release holds broader prospects for applications. The strategy of altering the optical properties of molecular structures to modify their light-controlled gas release behavior is of significant guiding importance for the design and synthesis of novel photosensitive GRMs molecular compounds.

Ultrasound can focus on local areas within the tissue (with a focal size of up to micrometers) and has a stronger tissue penetration (the tissue penetration depth of 1 MHz sound waves can reach 20 cm). Therefore, ultrasound-responsive gas-controlled release is an advantageous drug delivery method [115]. Research on the release of therapeutic gases using ultrasonic stimulation is currently focused only on liposome-type nanomedicines that encapsulate gases. Nanomedicines constructed using liposome microbubbles encapsulating gas molecules are crushed by cavitation of ultrasonic waves, thereby releasing gas molecules encapsulated therein. However, the disadvantages include low gas loading capacity, poor stability, inability to achieve controllable and uneven size particles [116].Therefore, developing a novel ultrasound-responsive nanomedicine with excellent therapeutic performance and achieving safe and controllable release of therapeutic gases in the body is currently a challenging problem in the research of acoustically responsive materials.

X-rays have a very high penetration rate in living organisms, and low-dose X-rays can trigger the release of gas. Highly penetrating X-rays can be used to precisely control the release of medication in deep-seated lesion areas. By adjusting the X-ray radiation dose and duration, on-demand gas release can be achieved [117]. The disadvantage of this system is that the gas release is poorly controllable, and in the absence of X-ray irradiation, NO is still released slowly and spontaneously, which is mainly due to the instability of the SNO prodrugs in the physiological environment. Therefore, how to improve the stability of X-ray responsive nanodrugs and the controllability of X-ray controlled release gases are important issues that need to be addressed.

### Endogenous responsive controllable release of gases

Endogenous stimulus-responsive controlled gas release primarily involves the utilization of specific microenvironments within the body, where nanomedicines release gas under the stimulation of these microenvironments. Compared to exogenous stimulation sources such as optical and acoustic stimulation, endogenous stimulation is a non-invasive, green way to mediate gas release, with the advantages of unlimited depth of penetration in the body and in situ targeted release without invading normal tissues and cells.

It has been found that spinal cord injuries and tumors are similar in that hydrogen peroxide (H_2_O_2_) levels are significantly higher than in normal cells, and thus a class of hydrogen peroxide-responsive nanomedicines can be designed to take advantage of the high H_2_O_2_ expression in tumor cells [118]. H_2_O_2_-responsive gas release primarily involves utilizing H_2_O_2_ in the microenvironment of spinal cord injuries to trigger the therapeutic gas release from nanomedicines, thereby rescuing neuronal cells. And one of the main approaches to achieve hydrogen peroxide-responsive gas release is to develop hydrogen peroxide-responsive gas prodrugs.

The mildly acidic environment of inflamed tissues can be controlled by acid-responsive gas release. An acid-degradable carrier, MSN-CaP, was used to load NO gas prodrugs and realize acid-responsive NO gas release [119]. The calcium phosphate (CaP) coating serves the purpose of protecting the loaded prodrug and facilitating acid-responsive dissolution. At lower pH, the CaP coating dissolves, exposing the loaded NO prodrug, 2-nitrobenzaldehyde (NBA), which spontaneously decomposes to generate NO gas. However, the highly unstable NBA prodrug may lead to instability of the nanomedicine. The key challenge that urgently needs to be addressed is how to achieve controlled gas release in a mild acidic microenvironment, ensuring the high stability of nano gas drugs under normal physiological conditions and preventing gas leakage.

### Targeted gas release

In order to eliminate the disadvantage of rapid free diffusion of gases, it is essential to realize targeted gas transport. By introducing targeted nanocarriers to load gas prodrugs, novel targeted nanogas drugs can be constructed for targeted transportation of gases. The targeting of nanomedicines can be achieved primarily through two approaches: passive targeting and active targeting. Passive targeting means that particles below 100 nm in size can passively accumulate in the spinal cord injury region through a tumor-like "enhanced permeation and retention" effect (EPR effect). Thus, in order to achieve targeted gas transport in spinal cord injury, the size of the nanomedicine should be kept below 100 nm.

Active targeting is mainly achieved through ligand recognition (such as chondroitin sulfate proteoglycan) and external field guidance (such as magnetic field targeting). By constructing a magnetic iron tetroxide-NORMs-silica nanoparticles, magnetically guided accumulation of nanoparticles under in vitro simulated conditions is achieved by a magnetic targeting strategy [120]. However, there are still few reports of targeted nanomedicines for gas therapy.

Gasotransmitters are highly membrane-permeable and can easily transmit signals by autocrine or paracrine means, and are the most active class of substances in the regulation of cellular homeostasis. Gas therapy is an emerging and highly promising strategy for anticancer treatment. The application of nanotechnology in the field of gas therapy will have far-reaching implications [121]. Nanogas therapy focuses on addressing two key scientific issues: controlled gas release and targeted gas transport. Controlled gas release and targeted gas transport are two critical factors in nano gas therapy. Although it is still difficult to carry out the implementation of nanomedicine into clinical trials, the future of its translation to the clinic is more promising.

## Prospects

There are special gasotransmitters such as H_2_S, NO, CO, O_2_, and H_2_. They play a role in regulating the nervous system, cardiovascular system, musculoskeletal system, and immune-endocrine system. At the same time, these gasotransmitters can also regulate various physiological functions of the human body system by specifically binding to multivalent transition metals [109, 122, 123], which has an important influence on the normal operation of human physiological processes and the effective regulation of pathological processes. Exogenous supplementation of such gasotransmitters has a significant therapeutic effect when the organism is in the midst of a major disease (e.g., inflammatory response, ischemic damage to organ tissues, cardiovascular disease, cancer, etc.) [109, 122, 123].

The use of gas therapy has provided new ideas for the treatment of various diseases, but its gaseous nature and potential toxicity have hindered its widespread clinical use. The perfect combination of nanomaterials and gasotransmitters optimizes the targeting of therapeutic gases to the site of injury and maintains therapeutic concentrations to harness their anti-inflammatory, antioxidant and tissue-protective effects. Gas therapy requires smarter synergistic control of the released nanomaterials to respond to different stimuli for various types of diseases. Designing multifunctional combined anti-inflammatory and antioxidant therapeutic platforms based on careful consideration of material design, construction, performance, and safety, the gaseous nanoplatforms will have a promising application.

How to prepare nanomaterials into multifunctional gas molecule donor materials to achieve the release of therapeutic gas molecules and metal ions with pro-neural vascular growth at the site of SCI, so as to achieve the dual functional effects of inhibiting inflammation and neurovascular regeneration. Promoting regeneration of axons and injured neuro-vessels after SCI are the goals of further researches to optimize therapeutic strategies for different types of SCI in the clinic.

## Data Availability

Not applicable.

## Abbreviations

SCI: Spinal Cord Injury
H_2_S: Hydrogen sulfide
NO: Nitric oxide
CO: Carbon monoxide
H_2_: Hydrogen
HBO: Hyperbaric oxygen
CBS: Cystathionine beta-synthase
CSE: Cystathionine gamma-lyase
3-MST: 3-Mercaptopyruvate sulfurtransferase
CAT: Cysteine aminotransferase
NaHS: Sodium hydrosulfide
TJ: Tight Junction
AJ: Adherens Junction
BSCB: Blood-Spinal Cord Barrier
Nrf2: Nuclear factor erythroid 2-related factor 2
Bcl-2: B cell lymphoma-2
IL-1: Interleukin-1
TNF-α: Tumor necrosis factor-α
NF-κB: Nuclear Factor Kappa-B
NPCs: Neural progenitor cells
NOS: Nitric oxide synthase
iNOS: Inducible nitric oxide synthase
cNOS: Constitutive nitric oxide synthase
nNOS: Neuronal nitric oxide synthase
eNOS: Endothelial nitric oxide synthase
siRNA: Small interfering RNA
HO: Heme oxygenase
HSP: Heat shock protein
Hb: Hemoglobin
COHb: Carboxyhemoglobin
cGMP: Cyclic guanosine monophosphate
CORMs: Carbon monoxide-releasing molecules
BDNF: Brain-derived neurotrophic factor
IRE1: Inositol-requiring enzyme 1
HBOT: Hyperbaric Oxygen Therapy
VEGF: Vascular endothelial growth factor
MMP-2: Matrix metalloproteinase-2
SOD: Superoxide dismutase
GPX: Glutathione peroxidase
MCP-1: Monocyte chemotactic protein-1
ROS: Reactive oxygen species
NCSS: Neurocervical spine scale
BMSC: Bone marrow mesenchymal stem cells
mitoK_ATP_: Mitochondrial ATP-sensitive potassium channels
GRMs: Gase Releasing Molecule
NIR: Near-infrared
H_2_O_2_: Hydrogen peroxide
CaP: Calcium phosphate
NBA: 2-Nitrobenzaldehyde
EPR: Enhanced permeation and retention
CSPG: Chondroitin sulfate proteoglycan
